# ‘On the surface’: a qualitative study of GPs’ and patients’ perspectives on psoriasis

**DOI:** 10.1186/1471-2296-14-158

**Published:** 2013-10-20

**Authors:** Pauline A Nelson, Zoë Barker, Christopher EM Griffiths, Lis Cordingley, Carolyn A Chew-Graham

**Affiliations:** 1 Dermatology Research Centre, Institute of Inflammation and Repair, The University of Manchester and Manchester Academic Health Science Centre, Suite 14, 5th Floor, Williamson Building, University of Manchester, Oxford Rd, Manchester M13 9PL, UK; 2 Manchester Medical School, The University of Manchester, Stopford Building, Oxford Road, Manchester M15 9PT, UK; 3 Dermatology Research Centre, Institute of Inflammation and Repair The University of Manchester and Manchester Academic Health Science Centre, Salford Royal NHS Foundation Trust, Irving Building, Manchester M6 8HD, UK; 4 Dermatology Research Centre, Institute of Inflammation and Repair, and Manchester Centre for Health Psychology, The University of Manchester and Manchester Academic Health Science Centre, 6th Floor, Williamson Building, University of Manchester, Oxford Rd, Manchester M13 9PL, UK; 5 Research Institute, Primary Care and Health Sciences, Keele University, Keele, Staffordshire ST5 5BG, UK

**Keywords:** Psoriasis, Qualitative research, Primary health care, Patient perspectives, Self-care, General practitioners, NICE guidelines

## Abstract

**Background:**

Psoriasis is a chronic, inflammatory skin disease affecting approximately 2% of the UK population and is currently incurable. It produces profound effects on psychological wellbeing and social functioning and has significant associated co-morbidities. The majority of patients with psoriasis are managed in primary care, however in-depth patient and GP perspectives about psoriasis management in this setting are absent from the literature. This article reports an in-depth study which compares and contrasts the perspectives of people with psoriasis and of GPs on the challenges of managing psoriasis in primary care.

**Methods:**

In-depth, qualitative semi-structured interviews were conducted with a diverse sample of 29 people with psoriasis and 14 GPs. Interviews were coded using principles of Framework Analysis to enable a comparison of patient and practitioner perspectives on key issues and concepts arising from the data.

**Results:**

Patients perceived GPs to be lacking in confidence in the assessment and management of psoriasis and both groups felt lacking in knowledge and understanding about the condition. While practitioners recognised that psoriasis has physical, emotional and social impact, they assumed patients had expertise in the condition and may not address these issues in consultations. This resulted in patient dissatisfaction and sub-optimal assessment of severity and impact of psoriasis by GPs. Patients and GPs recognised that psoriasis was not being managed as a complex long-term condition, however this appeared less problematic for GPs than for patients who desired a shared management with their GP incorporating appropriate monitoring and timely reviews.

**Conclusions:**

The research suggests that current routine practice for psoriasis management in primary care is mismatched with the expressed needs of patients. To address these needs, psoriasis must be recognised as a complex long-term condition involving exacting physical, psychological and social demands, co-morbidity and the development of new treatments.

General practitioners need to improve both their knowledge and skills in the assessment and management of psoriasis. This in turn will facilitate management of the condition in partnership with patients. Commissioning multi-disciplinary services, which focus on long-term impacts on wellbeing and quality of life, might address current deficits in care.

## Background

Psoriasis is a common, chronic and currently incurable skin disease affecting at least 2% of the UK population [1]. Many people live with psoriasis for the majority of their adult lives [2,3]. High levels of chronic physical, and psychosocial disability are reported [4-6], however the visible nature of psoriasis can be particularly disabling, with evidence of stigma, lowered self-image, depression, anxiety and suicidal ideation [7], even in mild psoriasis [8]. Co-morbidities include psoriatic arthritis [9], Crohn’s disease [10] and in severe psoriasis, an increased risk of cardiovascular disease (CVD) and of diabetes [11,12].

People are dissatisfied with the management of their psoriasis [13-17], and while recommendations have emphasised the need for assessment to include an objective evaluation of severity and extent as well as the patient’s subjective perception of impact [18], clinicians do not routinely assess the impact of the condition upon the individual [19]. Patients want their health care practitioners to acknowledge, in consultations, the stress and distress that often accompany living with psoriasis [20]. Additionally, low levels of medication adherence are problematic [21,22] and people may disengage from seeking help from within the health service due to dissatisfaction with care [20].

A recent collaboration between the Psoriasis Association of Great Britain and Ireland and the Mental Health Foundation [23] emphasises the need for people with psoriasis to understand that help is available to address its multi-faceted and long-term nature. In addition, the report suggests that the new National Health Service National Commissioning Board (NCB) should ensure that all local health economies establish clear referral pathways for psoriasis, with closely allied multi-disciplinary teams who can address the complex nature of this long-term condition (LTC). Long-term-conditions are increasingly important determinants of quality of life and health care costs in populations worldwide. Primary care is seen as the optimal context to deliver care for people with LTCs because it is accessible and efficient, with the emphasis on ‘shared management’ in which the general practitioner (GP) supports patients in the self-management of their condition [24].

The recently published National Institute for Health and Clinical Excellence (NICE) Clinical Guideline for the Assessment and Management of Psoriasis (CG 153) [25] identifies that most people with psoriasis are managed in primary care, although up to 60% may need referral to specialist services at some point in their lives. The current literature on healthcare practitioners’ perspectives of managing psoriasis is sparse and focuses on the views of dermatologists. Studies comparing dermatologists’ and patients’ views about disease severity, impact on quality of life and treatment goals report low levels of concordance [26-28] with dermatologists describing psoriasis as a “frustrating” condition due to its incurability [29].

The experiences of people affected by psoriasis in primary care or from the broader community have been reported only rarely in the literature. Furthermore, and despite the fact that most people with psoriasis will be managed in this setting [10] the views of GPs are also absent. This is of particular concern since GPs have a key role in identifying, monitoring and helping to reduce the risk of co-morbidities of psoriasis such as CVD and depression [30]. To address these gaps, qualitative interviews with GPs and patients were undertaken which enabled a comparison of views. This article reports an in-depth study of the perspectives of people with psoriasis and of GPs on the challenges of managing psoriasis in primary care.

## Methods

The study was an in-depth, qualitative interview study involving a community sample of people with psoriasis and GPs in the North West of England. Approval was obtained from the University of Manchester’s ethics committee for the patient and GP studies (Ref 10325 and Ref 11353 respectively) and from the Greater Manchester Primary Care Research Governance Partnership (ReGroup) for the GP study (Ref 2011/280). Qualitative interviews with patients and with GPs were conducted with prior informed written consent from all participants.

### Sampling and recruitment

Twenty-nine people with psoriasis were recruited by placing poster advertisements in community venues (libraries, community centres, places of religious worship, shops and post offices) in Greater Manchester and via the Psoriasis Association website (see Table 1 for advertisement wording).

**Table 1:** Study advertisement wording

**Headline information**	**Contact details**
**Coping with Psoriasis**	If you would like to be interviewed to express your views or if you would like to find out more please contact.
**Do you have psoriasis or know someone who has?**	(researcher) on:
	(contact details)
We would like to learn from you how psoriasis affects your life and what helps you manage it so that people with psoriasis can get better care. This research is funded by the NHS and is independent of any commercial interests.	We will arrange to see you at a time and place that is convenient and can come to your home. If you prefer to come to us we will pay your travelling expenses.

Twenty-six participants were from community sources and three from the Psoriasis Association membership list. Sampling was purposive for maximum variation [31], on characteristics of sex, age, self-identified ethnic/socio-economic background and self-identified severity of psoriasis, duration and treatment (assessed by asking participants to describe in their own words how severe they perceived their psoriasis to be both currently and in the past). Participants were interviewed at whatever location was most convenient to them (own home, workplace, community centre or university meeting room) over the spring of 2011. Interviews were audio-recorded and transcribed verbatim. This paper reports two of the themes developed in the qualitative analysis. Other findings based upon the patient interviews have been reported elsewhere [20]. Seven GPs in urban/inner city practices across each of three areas of Greater Manchester (north, central and south), UK (21 GPs in total) were emailed by CAC-G and invited to take part in qualitative interviews about psoriasis management. General practitioners from 14 of these practices were purposively sampled to achieve a mixed sample in terms of gender, age, ethnicity, ‘type’ of GP (eg. partner, salaried or trainee) and size of practice. Interviews were conducted over the summer of 2012.

### Data collection and analysis

Topic guides for both sets of interviews were developed from the relevant literature (see Table 2).

**Table 2:** Interview topic guides

**Guide**	**Topics**
**Patients**	Physical, emotional and social effects of psoriasis
	Self-care strategies
	Use of health services
	Relationships with health care professionals
**GPs**	Psoriasis management in primary care
	GPs’ self-appraisal of their own management of patients with psoriasis

Analysis began shortly after initial data collection, using an iterative coding procedure in accordance with principles of Framework Analysis, an analysis approach appropriate to applied health services research, enabling investigation of *a priori* issues while simultaneously allowing for identification of newly emergent ideas in the data [32]. For all interviews the progressing analysis informed subsequent data collection. Transcripts underwent preliminary coding by individual researchers with core themes subsequently analysed by the team using constant comparison within and across cases, with attention to disconfirming cases and possible reasons for differences. Sampling continued on both datasets until main data categories were saturated and no new insights were apparent [33]. Initial themes from the two datasets were emergent, but in development of the paper, comparisons were made across the datasets and secondary analysis was carried out based on the recently published NICE Guideline [25].

## Results

Ninety people with psoriasis responded to advertising and a varied sample of 29 was drawn to ensure diversity (Table 3). Of 19 GPs invited to participate, 14 agreed to be interviewed (Table 4).

**Table 3:** Patient demographics

**Characteristic**	**Female**	**Male**	**Totals**
	**(24–72 years)**	**(20–84 years)**	
	**14**	**15**	**29**
**Self-identified ethnicity**			
Bangladeshi	1	1	**2**
British Pakistani	0	1	**1**
Indian	1	4	**5**
Pakistani	2	0	**2**
Pakistani/European parents	1	0	**1**
White	9	9	**18**
**Socio-economic background**			
***Employment***			
Full-time work	3	7	**10**
Part-time work	3	3	**6**
Unemployed	4	1	**5**
Full-time study	2	3	**5**
Retired	2	1	**3**
**Self-identified psoriasis severity**			
Severe	1	0	**1**
Moderate-severe	3	6	**9**
Moderate	3	2	**5**
Mild-moderate	6	4	**10**
Mild	1	3	**4**

**Table 4:** GP demographics

**Characteristic**	**Female**	**Male**	**Totals**
	5	9	14
**Age**			
25–40	1	3	**4**
41–55	3	5	**8**
>55	1	1	**2**
**Self-identified ethnicity**			
White British	4	6	**10**
White other	0	2	**2**
Other	1	1	**2**
**GP type**			
GP partner	4	7	**11**
Salaried GP	0	1	**1**
GP trainee	1	1	**2**
**Practice size**			
≤6000 (small)	2	5	**7**
>6000 (large)	3	4	**7**

From the perspectives of GPs and patients, two broad themes, together with their sub-themes are presented. These themes build on our prior published work on patient perspectives [20], by comparing and contrasting patient perspectives with those of GPs and linking the findings to the recently published NICE Guideline (CG153) [25].

**Figure 1:**
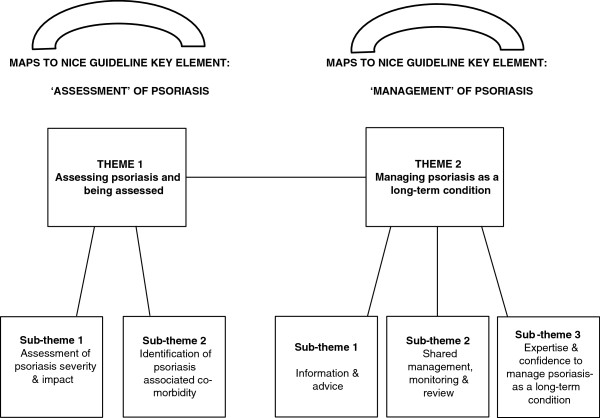
Analytic themes mapping to key NICE guideline elements ‘assessment’ and ‘management’ of psoriasis.

The two themes presented are: 1) Assessing psoriasis and being assessed, comprising views on the assessment of psoriasis severity, its perceived impact on patients and identification of psoriasis-associated co-morbidity and; 2) managing psoriasis as an LTC, comprising views on information and advice, shared management, monitoring and review and expertise and confidence. Broad themes one and two from the analysis map respectively to the dual elements of the recently published NICE Guideline [25], namely the ‘assessment’ and ‘management’ of psoriasis (see Figure 1 for a diagrammatic representation of the analytic themes and how they map to the NICE Guideline elements).

The two broad analytic themes are now discussed, with Illustrative data identified by patient or GP number to support the analysis.

### Assessing psoriasis and being assessed

There was variation in the disease models used by GPs with some recognising psoriasis as a complex systemic disease with a range of psychological, social and physical sequelae. However most GP accounts described less sophisticated clinical management approaches, reflecting a model of psoriasis as a straightforward skin condition:

‘I think of it primarily as a skin complaint…I am aware that there is…systemic problems…but, I think in my own mind, I sometimes find it difficult to put the two together’ (GP5)

By contrast, patients described psoriasis as a condition affecting more than just skin, with exacting physical, emotional and social consequences:

*‘It’s a skin condition but it goes* ***beyond*** *that. It doesn’t stay there. It sort of runs deeply…there was discomfort on me. It got to the stage where I was thinking about it so much I actually cancelled meeting my friends’* (P17)

#### ***Assessment of psoriasis severity and impact***

The perspectives of patients and GPs differed in relation to assessing the physical impact of psoriasis. Some GPs described barriers to physical examination, including feeling uncomfortable asking patients to undress, but also reluctance to examine a condition that could be perceived as unpleasant for both practitioner and patient. By contrast, patients wanted practitioners to engage in a more thorough examination and discussion about their condition:

*‘[GP] has never looked…never examined me for my skin…in six years or something like that’* (P8)

*‘I probably don’t examine them enough because I look at what they show me…so I’ve never even thought about the discussion…and well obviously I feel uncomfortable undressing…’* (GP3)

*‘It can be quite distressing both for the patient…with the kind of itching oozing scalp but it’s not the … most pleasant thing in the world…to examine either’* (GP6)

Some GPs acknowledged that psoriasis may have an adverse effect on people’s occupations and relationships, seeing psoriasis as an extra stressor for patients who have difficult lives already:

*‘I think there’s something quite stigmatising about psoriasis’ (*GP5)

*‘I think skin conditions, because they’re “on the surface”, produce an awful lot of worry in people’* (GP14)

*‘What makes it more difficult for* [patient] *is that actually sleeping with his wife is not very pleasant when he is really bad* [during a flare]*’* (GP2)

Patients concurred with these views by reporting that appearance-related relationship issues were often of most concern to them, however their accounts were often graphic and emotionally charged, highlighting the extent to which psoriasis could not only be bothersome but distressing and socially disabling:

*‘…people’s reactions,* ***girls’*** *reactions…it’s your self-consciousness of it. That could be quite overwhelming at points’* (P1)

*‘Imagine trying to be sat somewhere with your nails full of this stuff and it’s coming from your head and your face and, kind of, interacting with people is* ***really****, really hard’* (P20)

Despite recognition of the possible emotional and social effects of psoriasis, several GPs minimised its emotional impact, losing the opportunity for more focused assessment and discussion of wellbeing. By contrast, many patients described their frustration and disappointment that within consultations their GPs had not explored the wider impact of psoriasis:

*‘I haven’t got any patients who are distressed to the point of not wanting to go out, not wanting to do anything, where it impacts on their lives…mostly because I think we manage their psoriasis sufficiently well’* (GP10)

*‘You don’t really get a chance to express yourself. I’ve not been to hospital yet with* [psoriasis]*, so I don’t know if somebody is going to be able to listen to what is actually going on for me. I kind of, feel embarrassed going out. There’s no one listening to that’* (P20)

#### ***Identification of psoriasis-associated co-morbidity***

While some GPs identified that depression could be more common in patients with psoriasis, many reported that depression was so prevalent in all their population it was difficult to assess its relationship with the condition. Patients by contrast often referred to psoriasis as depressing:

*‘40% of our patients take an antidepressant…so should we just ask everyone [with psoriasis] “do you feel sad?”’* (GP2)

*‘I don’t know why but I got in this really deep depression…all the dry skin and it’s so dry…and I did feel…I went home and I couldn’t stand it’* (P14)

Most GPs were aware that psoriatic arthritis was a co-morbidity, but fewer recognised possible links with CVD risk. In common with depression, GPs did not routinely case-find for CVD, citing perceived low mortality and prevalence of psoriasis as a barrier to screening. Patients were commonly unaware of the possible CVD risk conferred by psoriasis:

*‘…the main aspect is that [patients] are pretty unlikely to die from psoriasis…it’s not going to predispose them to other major problems…unless they get psoriatic arthropathy’* (GP4)

*‘Not that I know of [co-morbidities other than psoriatic arthritis]. I’ve not been told anything, unless a surprise is waiting for me when I go to see the dermatologist’* (P16)

General Practitioners recognised that psoriasis could be a demanding condition with impact on patients’ emotional wellbeing and quality of life, however they were less likely to undertake structured assessments which incorporated broad discussions either about impact or possible co-morbidities.

### Managing psoriasis as an LTC

#### ***Information and advice***

General Practitioners recognised psoriasis as an LTC due to its chronicity, but its management was not comparable to that of other LTCs. While patients believed that information from their GPs about their condition was lacking, GPs suggested patients did not actively seek information about their psoriasis. Some commented that this may be an area they should address:

*‘I think patients often want to know more…than GPs tell them…so they probably want to know more than maybe they’re asking’* (GP12)

*‘[GP] did not give me information, or didn’t sort of think it was important’* (P18)

Many patients reported that they managed their psoriasis in isolation from their GP and in the absence of adequate information and advice, often with significant levels of uncertainty about prescribed treatments (or need for a different level of treatment). In contrast, some of the GPs stressed the patients’ responsibility for managing psoriasis treatments particularly in terms of persistence:

*‘I get fed up…you can use [creams] for a period of time but eventually they stop working…my psoriasis will keep growing and the cream will lose its effect. So I generally don’t put anything on my skin’* (P8)

*‘..so much of the success of psoriasis treatments is based on patients’ ability to manage it themselves and apply stuff appropriately…we can prescribe anything we like but if the patient isn’t using it correctly it’s probably not going to work’* (GP11)

#### ***Shared management, monitoring and review***

Patients described the need for continuity with their GP to plan management of their condition, but felt this was not offered. Conversely, GPs considered responsibility for re-attending to lie with patients, being undecided about whether patients wanted to be followed up:

*‘There was never any kind of follow-up to it and I never went to the GP for any other reason, over the 10-year period’* (P11)

*‘Patients’ willingness and desire to be regularly reviewed is very variable. A lot of them, even if you say come back in a month…they don’t come back, they don’t really want to see you again’* (GP8)

Some patients reported that their own knowledge and experience were not explored or valued by practitioners, with limited opportunity to negotiate management plans:

*‘GPs, will do as much as they can with what they're allowed to give you, but if it doesn't work then it's just a case of…“well, that's all that's available”’* (P16)

This corresponded with the GP accounts. There was little evidence that on-going, shared management in psoriasis was something they did routinely. Only one GP raised the issue of negotiating treatment goals with patients:

*‘Perhaps I don’t ask enough about what their treatment goals are…we all have our own assumptions about what a good outcome is and we don’t necessarily share that with patients’* (GP5)

For LTCs that are included in the UK Quality and Outcomes Framework (QOF) [34], recall systems operate to invite patients for regular review. Psoriasis is currently not included in QOF, and GPs reported that review and recall were not organised for people with psoriasis:

*‘The difference* [compared to other LTCs] *is that our systems will pick up a lot of the other ones…and we’ll end up chasing them…because of the way we’re paid through QOF…we’ve got an incentive to manage a certain group of chronic diseases but with other ones there isn’t the same kind of incentives…so you don’t get the systems in to …chase them’ (*GP7)

This discrepancy did not go unnoticed by patients with other conditions and for whom this apparent lack of attention to their psoriasis was a source of dissatisfaction:

***‘Big*** *difference* [between psoriasis care and other LTC care]…*because of my heart problems and the medication I’m on, they’ve given me a lot of time’* (P20)

Many patients were consequently not in regular contact with their primary care providers and were often coping sub-optimally with their psoriasis, believing that little could be done to help. By contrast, GPs often assumed that when patients did not return for review, they were not experiencing problems but rather were coping well:

*‘It’s depressing…you can’t see it getting any better…just worse and worse and worse…might be a slight improvement, but the general trend…it is very depressing’* (P1)

*‘I think some* [patients] *just think “oh well, it’s not so bad at the moment I won’t bother”…or some of them just get repeat prescriptions for something they like’* (GP8)

Within consultations, addressing lifestyle issues in relation to psoriasis triggers, exacerbation or increased CV risk did not appear to be prioritised by GPs. With the exception of alcohol, GPs referred to few lifestyle factors as important in psoriasis management. Patients too suggested that discussion of lifestyle did not routinely take place in consultations with GPs:

*‘Alcohol can make [psoriasis] worse but not much else’* (GP12)

*‘I’ve had pretty much no advice other than – let’s try this treatment. So no lifestyle advice or anything like that’* (P1)

#### ***Expertise and confidence to manage psoriasis as an LTC***

Most GP accounts indicated low levels of expertise and confidence in the management of people with psoriasis, blaming lack of undergraduate and postgraduate training in dermatology. Patients also perceived GPs to be lacking in knowledge and expertise:

*‘There wasn’t a huge amount of dermatology* [training]…*I would probably do more if I knew more’* (GP9)

*‘GPs just basically didn’t know what to do with it because they don’t come across it, or if they do come across it they have limited knowledge of it’* (P6)

Some GPs deferred to their patients as having greater knowledge, and viewed them as experts in their condition, particularly in terms of their experiences of treatments tried and failed:

*‘People with psoriasis are experienced, they know about their illness…it’s no good saying “here’s a treatment” if they say “yeah, I tried that and I really hated it” or “it smelled”’ (*GP8)

In contrast, several of the patient accounts referred to low levels of confidence or knowledge about psoriasis, particularly in terms of causes, triggers, how to manage symptoms, and wanting expert advice:

*‘I don’t know what causes it to be like this. I have oily fish, I eat fish twice* aweek*. I try to eat healthy. I use the creams which they say. Nothing seems to calm it down. It just flares up and that’s it. Over the years it has got worse and what’s going to happen in a bit?’* (P12)

General Practitioners acknowledged difficulties managing a condition that did not seem to respond to their input. This mirrored the accounts of patients who believed there was little a GP could do to help with psoriasis and were consequently reluctant to consult:

*‘[Patient] does have this chronic condition that’s been very difficult to manage over the years and … I often feel a little bit helpless trying to … help’* (GP6)

‘*I should have gone to my GP about* [psoriasis], *but…I was just under the general impression that there was very little they could do’* (P11)

General Practitioners and patients concurred on the perceived lack of shared management of psoriasis and that the condition was not subject to the same level care as other LTCs. Specifically, shared management of patients’ needs for information and support for medications management, self-care and coping was problematic. Systems for adequate monitoring and review were lacking.

## Discussion

### Summary of main findings

This is the first article to present the perspectives of people with psoriasis alongside those of GPs about management of the condition in primary care. While patients may sense practitioners’ lack of confidence in the assessment and management of psoriasis and GPs may mistakenly assume that patients possess expertise in their condition, *both* feel that they are lacking in knowledge and understanding of psoriasis. Practitioners recognise that psoriasis may have physical, emotional and social impacts but report circumventing discussion of these in consultations, which may result in sub-optimal assessment of severity and impact. In contrast, patients reported them as disabling and would welcome opportunities to discuss them. Patients and GPs concur on the lack of management of psoriasis as an LTC, however this appears less problematic for GPs than for patients, who desire appropriate review and monitoring.

### Comparison with previous literature

The previous literature demonstrates that psoriasis is demanding to live with [20,35] and that dissatisfaction with care exists among patients [15,20] whose views on psoriasis management may differ from those of dermatologists [28,29]. Our study contributes in-depth accounts that highlight the contrasts in the perspectives of psoriasis patients and GPs and suggests reasons for the low number of primary care consultations by patients [36]. This research offers a more nuanced understanding of the mismatch between practitioner and patient views, highlighting factors which may contribute to patients’ perceived difficulties with understanding and appropriately managing their psoriasis and the lack of shared management of psoriasis as an LTC in primary care [37].

The 2012 NICE Guideline for psoriasis [25] recognises primary care as the context in which the vast majority of psoriasis patients are managed, stressing the significant impact that all aspects of psoriasis have on wellbeing. This underlines the need to adopt a broader approach to the management of psoriasis which integrates physical and emotional approaches to care. Such an approach would include adequate assessment of all aspects of the condition, comprising objective assessment of the extent and severity, as well as the patient’s own perception of impact, prompt, effective treatment and long-term disease control including discussion of lifestyle support and appropriate referral. The narrow view expressed by some GPs in this study suggests that there are gaps in knowledge, skills and training that need to be addressed.

### Strengths and limitations

The patient sample was diverse. Recruiting from the community may have enabled participants to give more candid accounts of their relationships with professionals. This could however, lead to a self-selection bias with the most dissatisfied people responding to advertising. The practitioner sample was relatively small, most being white British GP partners, and may not be representative of the views of GPs in the UK as a whole. However the data illustrate an important perspective on the management of psoriasis in primary care, which is currently absent from the literature. Data analysis benefited from the perspectives of professionals from a breadth of backgrounds including dermatology, primary care, health psychology and health services research [38].

### Implications for clinical practice and future research

Understanding and recognition of psoriasis as a complex LTC involving significant psychosocial impact, co-morbidity and the development of new treatments [39] has so far not extended to primary care where current routine practice is misaligned with the expressed needs of patients. The study was conducted before the NICE Guideline [25] was published and this of itself may raise GPs’ awareness of the complexity of psoriasis and the need to assess and manage psoriasis patients as they would do those with other LTCs. Thus, they would develop systems for on-going monitoring and management [40]. Psoriasis care is currently not incentivised under QOF [34], limiting the likelihood of GPs setting up disease registers and recall systems for psoriasis [41]. In addition, viewing psoriasis as a simple condition means that GPs may not provide patients with adequate opportunities to discuss their concerns. Experiences of such consultations may recursively discourage consulting about psoriasis, with adverse consequences for self-care and coping including adherence to prescribed medications. Better knowledge, understanding and training about psoriasis among GPs is needed, as well as improved consultation skills to enable management of the condition in partnership with patients.

## Conclusion

The importance of routine assessment of psoriasis in primary care was highlighted in recent guidance [25]. General practitioners should systematically record levels of psoriasis severity which contain an indicator of impact of the condition on their patients. Furthermore, the recent report by the Mental Health Foundation in collaboration with the Psoriasis Association [23] emphasises the need for multi-disciplinary services which address long-term impact on psychological wellbeing and quality of life. The challenge facing the NCB and Clinical Commissioning Groups will be to configure services which recognise psoriasis as a LTC, requiring systems of monitoring and integrated bio-psychosocial support similar to other LTCs, and with appropriate referral pathways for patients when more specialist help is needed. The current downward pressure on referrals as operationalized by the Quality and Productivity indicators in QOF [42] may act as a barrier to the development of such pathways for psoriasis if it continues to be seen primarily as a skin condition.

## Abbreviations

CVD: Cardiovascular disease; LTC: Long-term condition; NCB: National Commissioning Board; NICE: National Institute for Clinical Excellence; GP: General practitioner; QOF: Quality and outcomes framework.

## Competing interests

The authors declare that they have no competing interests.

## Authors’ contributions

PAN contributed to the design and analysis of both the patient and GP studies, collected the patient data, and drafted / revised the manuscript. ZB collected and co-analysed the GP data. CEMG acquired funding for the research programme and revised the manuscript for intellectual content. LC acquired funding for the research programme, conceived of the patient study, contributed to its design and analysis and revised the manuscript for intellectual content. CAC-G acquired funding for the research programme, conceived of both the patient and GP studies, contributed to their design and analysis and drafted the paper. All authors read and approved the final manuscript.

## The IMPACT Team

The Identification and Management of Psoriasis Associated Co-Morbidity (IMPACT) Team.

## Pre-publication history

The pre-publication history for this paper can be accessed here:

http://www.biomedcentral.com/1471-2296/14/158/prepub
